# Assessment of Albumin-Incorporating Scores at Hepatocellular Carcinoma Diagnosis Using Machine Learning Techniques: An Evaluation of Prognostic Relevance

**DOI:** 10.3390/bioengineering11080762

**Published:** 2024-07-28

**Authors:** Miguel Suárez, Pablo Martínez-Blanco, Sergio Gil-Rojas, Ana M. Torres, Miguel Torralba-González, Jorge Mateo

**Affiliations:** 1 Gastroenterology Department, Virgen de la Luz Hospital, 16002 Cuenca, Spain; 2 Medical Analysis Expert Group, Instituto de Investigación Sanitaria de Castilla-La Mancha (IDISCAM), 45071 Toledo, Spain; 3 Medical Analysis Expert Group, Institute of Technology, Universidad de Castilla-La Mancha, 16071 Cuenca, Spain; 4 Internal Medicine Unit, University Hospital of Guadalajara, 19002 Guadalajara, Spain; 5 Faculty of Medicine, Universidad de Alcalá de Henares, 28801 Alcalá de Henares, Spain; 6 Translational Research Group in Cellular Immunology (GITIC), Instituto de Investigación Sanitaria de Castilla-La Mancha (IDISCAM), 45071 Toledo, Spain

**Keywords:** mortality, hepatocellular carcinoma, Albumin-Bilirubin, eXtreme Gradient Boosting

## Abstract

Hepatocellular carcinoma (HCC) presents high mortality rates worldwide, with limited evidence on prognostic factors at diagnosis. This study evaluates the utility of common scores incorporating albumin as predictors of mortality at HCC diagnosis using Machine Learning techniques. They are also compared to other scores and variables commonly used. A retrospective cohort study was conducted with 191 patients from Virgen de la Luz Hospital of Cuenca and University Hospital of Guadalajara. Demographic, analytical, and tumor-specific variables were included. Various Machine Learning algorithms were implemented, with eXtreme Gradient Boosting (XGB) as the reference method. In the predictive model developed, the Barcelona Clinic Liver Cancer score was the best predictor of mortality, closely followed by the Platelet-Albumin-Bilirubin and Albumin-Bilirubin scores. Albumin levels alone also showed high relevance. Other scores, such as C-Reactive Protein/albumin and Child-Pugh performed less effectively. XGB proved to be the most accurate method across the metrics analyzed, outperforming other ML algorithms. In conclusion, the Barcelona Clinic Liver Cancer, Platelet-Albumin-Bilirubin and Albumin-Bilirubin scores are highly reliable for assessing survival at HCC diagnosis. The XGB-developed model proved to be the most reliable for this purpose compared to the other proposed methods.

## 1. Introduction

Hepatocellular carcinoma (HCC) is the primary liver tumor, accounting for approximately 80% of tumors affecting this organ [1]. Globally, it stands as a leading cause of cancer-related mortality. HCC ranks as the sixth most common cancer and the third leading cause of cancer-related deaths, according to recent data [2]. The epidemiology of this tumor has shifted since 2000 due to a decline in cases related to viral hepatitis (primarily hepatitis B and C virus), transitioning to a higher proportion of cases associated with Non-alcoholic Fatty Liver Disease (NAFLD) [3,4], more recently rebranded as Metabolic-Associated Steatotic Liver Disease (MASLD) [5]. This shift has transformed what was initially a decline in the incidence of this tumor due to hepatitis B vaccination, new disease-targeting medications, and the emergence of direct-acting antivirals against hepatitis C, to levels that have remained stable for years due to the global epidemic of NAFLD [6,7].

Most diagnoses of HCC occur in cirrhotic livers, with cirrhosis being the primary risk factor for its development [8,9,10]. This condition often delays the diagnosis of the disease as it is asymptomatic. Furthermore, it exacerbates the prognosis, as the patient’s baseline condition worsens due to the disease stage [11]. As evident in well-known scores such as the Child-Pugh-Turcotte score [12], albumin emerges as a critical factor that requires consideration. This protein is primarily synthesized in the liver and is linked to nutritional status, liver function, and regulation of oncotic pressure in blood vessels [13,14]. Patients with cirrhosis typically exhibit lower levels of albumin due to hepatic fibrosis. These levels correlate with poorer liver function and an increased risk of decompensation and complications associated with cirrhosis [15].

There is a growing body of evidence regarding risk factors for the development of HCC, and research is also focusing on new medications [16]. However, there remains a scarcity of evidence concerning prognostic factors at the time of HCC diagnosis. Investigating this aspect is crucial as it can determine the utility of the available therapeutic arsenal and enable personalized management of each patient based on their characteristics. The significance of albumin levels at this point is crucial. Such is the case that the new 2022 Barcelona Clinic Liver Cancer (BCLC) classification incorporates Child-Pugh levels, Model for End-stage Liver Disease (MELD), and also incorporates the Albumin-Bilirubin (ALBI) score into patient prognosis [17]. Considering this scientific evidence, the following study is proposed to evaluate the utility of ALBI, Platelet-Albumin-Bilirubin (PALBI), C-Reactive Protein (CRP)/Albumin and Child-Pugh score at the time of HCC diagnosis in terms of survival. Additionally, the study will assess the utility of other scores commonly used in these patients, such as the Eastern Cooperative Oncology Group-performance status (ECOG-PS) or MELD classification.

For this purpose, the implementation of Machine Learning (ML) techniques has been chosen. These techniques have been previously utilized in the medical field due to their capacity for analyzing a vast amount of variables, enabling the detection of patterns not typically detectable through conventional statistics [18]. There is already existing experience in the field of hepatology regarding these techniques [19,20], among others specialties in medicine, such as Oncology [21], Cardiology [22] or Internal Medicine [23]. Specifically, the eXtreme Gradient Boosting (XGB) algorithm is proposed as the reference method. The novel approach of our study lies in the application of the XGB machine learning algorithm for predicting mortality in HCC patients. Unlike traditional methods, which often rely on static and linear models, XGB leverages advanced techniques like parallel and distributed computing, regularization, and automatic handling of missing values. This allows for more accurate and generalizable predictions by efficiently managing non-linear relationships and complex data structures [24,25]. 

## 2. Materials and Methods

### 2.1. Population

A multicenter retrospective cohort study was conducted between Virgen de la Luz Hospital in Cuenca and the University Hospital of Guadalajara. This study encompassed all patients diagnosed with HCC between January 2008 and December 2022. Inclusion criteria for the study involved patients aged 18 years or older diagnosed with HCC. Diagnosis was established using imaging techniques or histological diagnosis. Patients initially diagnosed at another facility and those diagnosed at these hospitals but with unavailable variables for the proposed study were excluded.

### 2.2. Data Variables

To conduct the study, three types of variables were chosen:

Firstly, variables related to demographic data were selected. These included gender, age at HCC diagnosis, and the date of censorship or death if it occurred. Additionally, variables associated with patient comorbidities were collected. These encompassed smoking status, alcohol consumption, obesity, the presence of Diabetes Mellitus (DM), and dyslipidemia (DL). These variables were obtained from patients’ medical records. The ‘smoker’ variable was divided into never smoked or former smoker, and active smoker at the time of diagnosis [26]. Alcohol variable was defined based on criteria for alcohol abuse [27]. Obesity was defined by a Body Mass Index (BMI) ≥ 30 kg/m^2^ [28]. The diagnoses of DM and DL were established according to clinical guidelines or based on the presence of a previously established diagnosis in the medical history or the patient being under medical treatment [29,30].

Furthermore, variables related to the patient clinical condition and the diagnosis of HCC were collected. Those selected included the primary etiology of HCC (alcohol, HCV, HBV, NAFLD, hemochromatosis, autoimmune hepatitis, primary biliary cholangitis...), the presence of cirrhosis, and the Child-Pugh stage if cirrhosis was present. Additionally, data were collected on whether patients were diagnosed within an HCC screening program, the diagnostic method (imaging techniques or biopsy requirement for diagnosis), and the presence of clinically significant portal hypertension, emphasizing the presence of encephalopathy and ascites as the most common manifestations of decompensation [31,32]. Within this section, Eastern Cooperative Oncology Group-performance status (ECOG-PS) [33], MELD, number of lesions, size of the largest lesion (cms), presence of portal vein thrombosis, as well as metastasis and pathological lymph nodes were recorded. Using this data, BCLC stage was also calculated [17]. 

Finally, analytical variables deemed most useful for the study were collected along with others considered potentially influential in these patients’ prognosis. These included hemogram and coagulation data such as platelet count (10^3^/dL), lymphocytes (cells/mm^3^), neutrophils (cells/mm^3^), and the International Normalized Ratio (INR). Biochemical variables encompassed creatinine (mg/dL), sodium (mEq/dL), albumin (g/dL), bilirubin (mg/dL), Aspartate Aminotransferase (AST) (U/L), Alanine Aminotransferase (ALT) (U/L), alpha-fetoprotein (AFP) (ng/mL), and C-Reactive Protein (CRP) (mg/L). Consequently, calculations were performed for ALBI [34], Platelet-albumin-bilirrubin score (PALBI) [35] y CRP/albumin [36].

### 2.3. Machine Learning

In this study, the eXtreme Gradient Boost (XGB) method has been proposed as a predictive model because XGB is a widely used machine learning algorithm known for its high performance and accuracy. This system employs efficient parallel and distributed computing algorithms that significantly accelerate the training process, enabling the handling of large volumes of data swiftly. It offers high precision in predictions due to its capability to manage non-linear relationships and its use of regularization techniques (L1 and L2) that prevent overfitting, thereby improving the model’s generalization ability. Furthermore, it is highly flexible, supporting various loss objectives and allowing the customization of loss functions according to the specific needs of the problem. XGB automatically handles missing values, making it robust against incomplete datasets. It incorporates advanced techniques for managing categorical variables and selecting the most important features, and provides useful tools for model interpretation, such as feature importance and decision tree visualizations. It offers a wide range of adjustable hyperparameters to optimize the model’s performance and includes early stopping techniques to prevent overfitting and optimize training time [25,37]. XGB consistently outperforms other algorithms in accurately solving various data science problems [38,39,40]. Additionally, a comparative analysis was conducted with other supervised ML systems.

Considering a dataset S = x_j_, y_j_, the XGB model was formulated using the following:

(1) $$\hat{y_{j}}=\sum_{p=1}^{P}t_{p}\left(x_{j}\right)\;$$

where *x_j_* represents the input vector with *m* time variables, $\hat{y_{j}}$ denotes the predicted output, *y_j_* shows the output, *t_p_* represents a tree with leaf weight *w_p_* and structure *u_p_*, j = 1; 2;...; *n*, and *P* corresponds to the number of trees.

The formulated objective function for the proposed method is expressed in Equation (2). Employing a second-order Taylor expansion is integral to improving prediction accuracy in approximating the XGB objective function [41].

(2) $$R=\sum_{j}r\left(\hat{y_{j}},y_{j}\right)+\sum_{p}\Psi \left(t_{p}\right)$$

In Equation (3), *f_p_* represents the number of leaves on the tree. The R () function penalizes the complexity of the method. The learning rate is denoted by *λ* and *w_p_* is the vector of leaf scores. To control the complexity weight of the system, a parameter *γ* is employed. The aim is to optimize Equation (2) [42].

(3) $$\Psi \left(t_{p}\right)=\lambda f_{p}+\frac{1}{2}\gamma {\left\|\omega_{p}\right\|}^{2}$$

In this study, the proposed XGB method was compared with five widely used ML algorithms in the scientific community to evaluate its performance. These include Decision Trees (DT) [43], Gaussian Naive Bayes (GNB) [44], Bayesian Linear Discriminant Analysis (BLDA) [45], K-Nearest Neighbors (KNN) [46], and Support Vector Machines (SVM) [47]. We built the models using the MatLab Statistical and Machine Learning Toolbox (MatLab 2023a; The MathWorks, Natick, MA, USA). The dataset was split into two parts, with 70% used for training and the remaining 30% for testing, ensuring that patient information was not shared between the sets. To validate the results and prevent overfitting, we conducted 5-fold cross-validation. 

Optimizing the ML algorithms involves adjusting various hyperparameters during the training phase. Bayesian techniques were employed in this study to determine optimal hyperparameter values. This optimization method significantly improves the outcomes of the developed methods. Table 1 shows the main hyperparameters of the machine learning algorithms evaluated in the study.

**Figure 1:**
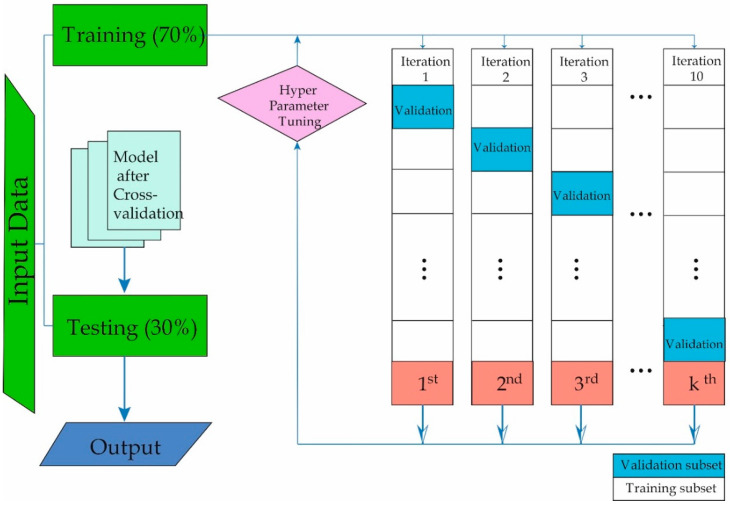
Representation of how the Machine Learning training and validation process was carried out.

Throughout all simulations, 100 iterations were executed to derive mean and standard deviation values in a uniformly random manner. This systematic approach mitigates the impact of noise, facilitating the calculation of relevant values and ensuring the attainment of statistically valid results [48]. The procedural phases employed in this study are delineated in Figure 1. Initially, subjects for study were selected, followed by the implementation of the database, and subsequent training and validation of ML methods.

Finally, the parameters checked to measure performance are:

(4) $$Recall\left(\%\right)=\frac{TP}{TP+FN}\times 100,$$

(5) $$Specificity\left(\%\right)=\frac{TN}{TN+FP}\times 100,$$

(6) $$Precision\left(\%\right)=\frac{TP}{TP+FP}\times 100,$$

(7) $$Accuracy\left(\%\right)=\frac{TP+TN}{TP+TN+FP+FN}\times 100.$$

In these equations, TP indicates the number of positive cases, TN is the true negatives, FN the false negatives and FP indicates the false positive cases.

In addition, the $F_{1}$ score and Matthew’s correlation coefficient (MCC) were employed during the study. The $F_{1}$ score is defined as:

(8) $$F_{1}\;score\left(\%\right)=\frac{Precision\cdot Recall}{Precision+Recall}\times 100$$

and the MCC [49], that measures the overall model performance, is described as:

(9) $$MCC\left(\%\right)=\frac{TP\cdot TN-FP\cdot FN}{\sqrt{\left(TP+FP)(TP+FN)(TN+FP)(TN+FN\right)}}\times 100.$$

Lastly, two additional metrics assessing the overall model performance, namely Cohen’s Kappa (CK) and degenerated Younden’s index (DYI) [49], have also been included in the study.

## 3. Results

After searching for patients, a total of 191 patients were eventually included in the study cohort. Out of the total, 4 were excluded for not meeting the inclusion criteria for the proposed study. The study included 25 women (13.4%) and 162 males (86.6%).

**Figure 2:**
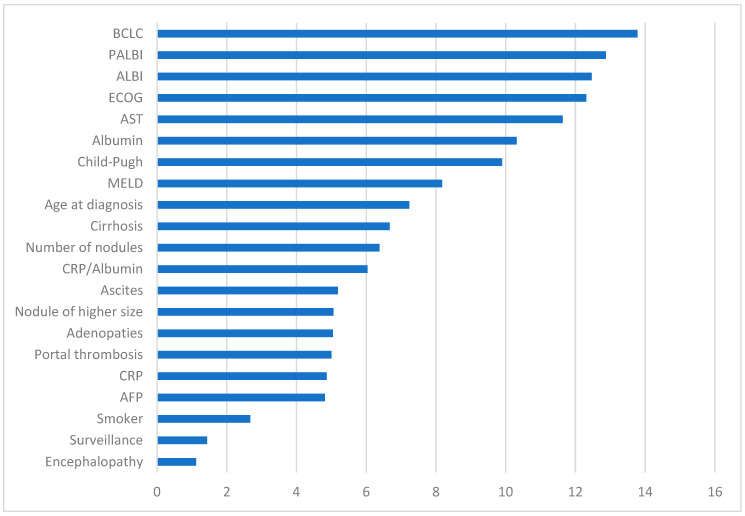
Summary of the predictive model results. The scores obtained for the most important analyzed variables are reflected. The *x*-axis represents the score of each variable, such that the higher the score, the greater the weight and importance within the predictive model. BCLC: Barcelona Clinic Liver Cancer; PALBI: Platelet-Albumin-Bilirubin; ALBI: Albumin-Bilirubin; ECOG: Eastern Cooperative Oncology Group; AST: Aspartate Aminotransferase; MELD: Model for End-stage Liver Disease; CRP: C-Reactive Protein; AFP: Alpha-fetoprotein.

Figure 2 depicts the importance ranking of the analyzed variables within the predictive model developed by the XGB algorithm. As shown, the most crucial variable was the BCLC criteria, closely followed by PALBI and ALBI scores. After these, the ECOG scale emerged as the most significant variable, followed by isolated analytical variables AST and albumin. Subsequently, the degree of liver function and functional reserve measured through Child-Pugh and MELD scores exhibited the most substantial influence. As evident, CRP/albumin levels, while not negligible, are positioned lower in the ranking, akin to tumor-dependent variables. Other factors with some weight were included in the final representation. Noteworthy is the role of AFP and screening in predicting mortality.

The results for the analyzed metrics of each proposed algorithm are detailed in Table 2. The parameters analyzed were balanced accuracy, recall, specificity, and precision. As can be appreciated, XGB achieves the highest values for these metrics. These values are approximately more than 6% higher across all these metrics compared to the second-performing method, KNN. These gaps become significantly wider when compared to the method that performed the worst, GNB, with differences close to 20%. On closer examination, concerning accuracy, the difference between XGB and KNN stands at a 6.41% performance advantage for XGB.

To assess the performance of the analyzed methods, several statistical metrics were used. The selected parameters have been employed and validated in multiple scientific publications. These include the Area Under the Curve (AUC) [50], F1 score [51], Matthews Correlation Coefficient (MCC) [52], Youden’s index (DYI) [53] and Kappa index [54]. For these parameters, the advantages of XGB remain around 6%. Notably, MCC values should be highlighted. Although there is a 5.49% difference between XGB (83.37%) and KNN (77.88%), MCC is considered one of the most reliable statistical indices. It reaches high values only when the model has been appropriately constructed within the parameters of the confusion matrix [55]. As can be observed, all these metrics are reflected in Table 3.

**Figure 3:**
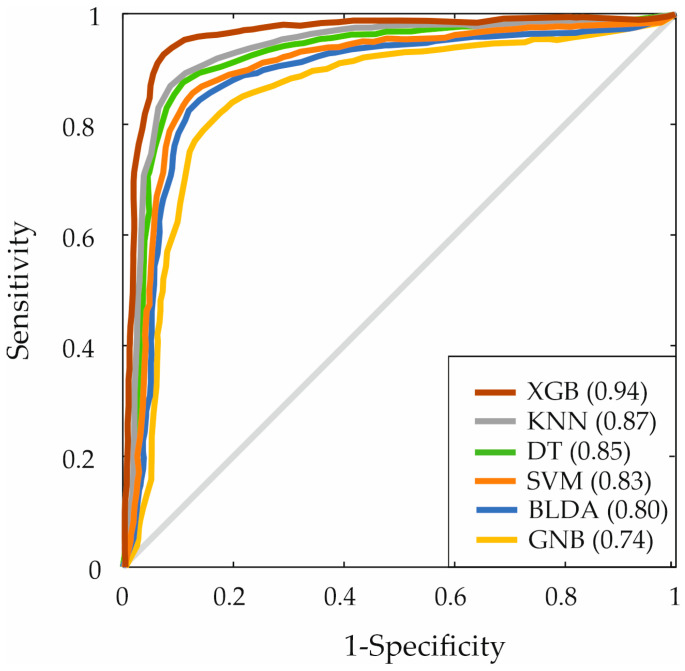
ROC curves of the algorithms. ROC: Receiver Operating Characteristic. SVM: Support Vector Machine. BLDA: Bayesian Linear Discriminant Analysis. DT: Decision Tree. GNB: Gaussian Naïve Bayes. KNN: K-Nearest Neighbors. XGB: eXtreme Gradient Boosting.

To represent the classification ability of each algorithm concerning the study objective, Receiver Operating Characteristic (ROC) curves were plotted (Figure 3). These curves result from the combination of sensitivity and specificity for each model [56]. The AUC (Area Under the Curve) for the XGB method shows the largest area (94%), being the only algorithm surpassing 90%. As can be appreciated, the rest of the systems (DT, KNN, BLDA, SVM) range between 80% and 87%, while GNB lags behind with 74%. This indicates that the model developed by XGB achieves higher accuracy in predicting the survival prognosis of patients at the diagnosis of HCC with the different analyzed variables.

**Figure 4:**
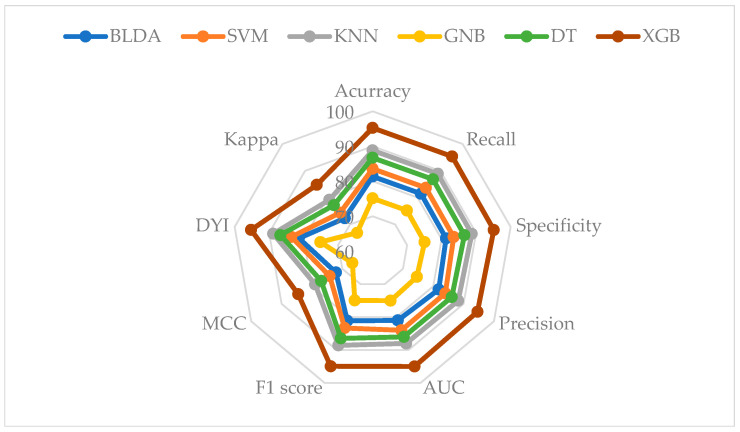

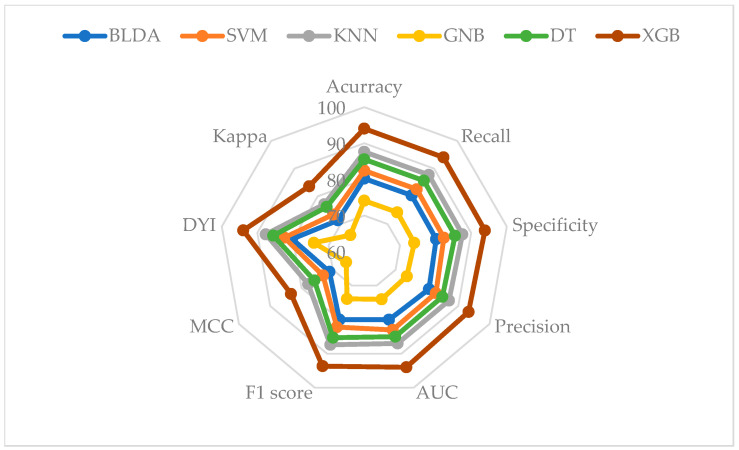
Image of the radar plot generated. Top: the training phase is represented; bottom: the same applies to the testing phase. SVM: Support Vector Machine. BLDA: Bayesian Linear Discriminant Analysis. DT: Decision Tree. GNB: Gaussian Naïve Bayes. KNN: K-Nearest Neighbors. XGB: eXtreme Gradient Boosting. AUC: Area Under the Curve. MCC: Matthews Correlation Coefficient. DYI: Youden’s Index.

Finally, to visually represent all the analyzed metrics, a radar plot was generated in Figure 4. A larger area indicates better predictive capability. At the top, all values for the different analyzed algorithms in the training phase are represented. At the bottom, the same process was conducted for the testing phase. As can be observed, the area for XGB is almost identical in both phases. This indicates that the model does not exhibit overestimation or underestimation, signifying the absence of overfitting. The implication of this is that the model is highly generalizable, and thus, a new input will generate an appropriate output. In contrast, the rest of the methods display a smaller area, rendering them less reliable for classifying these patients.

## 4. Discussion

Albumin is a vital protein for the proper functioning of human physiology. It is exclusively synthesized in the liver, producing around 15 g/day. If needed, the liver also has the capacity to synthesize double these amounts [57]. Its production is influenced by nutritional, hormonal, and inflammatory factors. In hepatic conditions, albumin serves as an excellent marker for liver function in patients with chronic liver disease and cirrhosis. Additionally, its half-life (14–21 days) can distinguish acute cases of liver failure if there is an underlying unknown previous liver damage. Care should be taken in interpreting it in cases of decompensated cirrhosis with ascites due to changes in volume distribution [14,58]. 

Currently, albumin is a topic of great interest in hepatology. There is significant evidence supporting its benefits, especially in managing ascites decompensation and refractory ascites [59,60]. Not only that, but multiple indices related to liver function are increasingly being employed. These scores have been associated with the survival of patients with liver diseases. In addition to the latest BCLC update, which now begins to take into account the ALBI score [17], there are numerous articles discussing the usefulness of these scores in different situations. For instance, in the study published by Oikonomou et al., the utility of ALBI and PALBI regarding the outcomes of patients with stable decompensated cirrhosis is assessed, determining that poorer values for both indices are associated with worse survival and a higher incidence of liver-associated complications [61]. In the study by Elshaarawy et al., the usefulness of the PALBI score as a predictor of variceal bleeding in patients with cirrhosis is evaluated, concluding that it is a good marker for rebleeding and mortality in such patients [62]. Meanwhile, in the article published by Ieda et al., the utility of different scores involving albumin (CRP/albumin, ALBI) as prognostic factors for mortality in patients with terminal cancer was evaluated. It was found useful in predicting mortality in patients with a life expectancy of less than two weeks, aiding in medical decision-making in such situation [63].

In the conducted study, among the variables related to albumin, PALBI exhibited the highest values, with ALBI levels almost at the same level. While not far behind, both scores showed a slightly lower value compared to the BCLC strategy. These slight advantages in favor of the BCLC model can be explained by the inclusion of ALBI within it, along with other variables. However, these results raise the question of whether the BCLC classification outcomes could improve by incorporating PALBI instead of ALBI for categorizing HCC patients, even though the difference between the two albumin scores is minimal. Furthermore, it also justifies the well-established utility of BCLC classification as a diagnostic, prognostic, and therapeutic reference in various clinical guidelines [8,9]. Nonetheless, the slight disparity between BCLC and these two scores implies that PALBI and ALBI are a highly reliable score for predicting mortality in patients diagnosed with HCC. They could be considered an alternative to the BCLC score for this purpose, for example, when tumor staging cannot be accurately determined.

The next evaluated score, although at some distance, was Child-Pugh. Its significance within the predictive model was substantial. This indicates that, despite possibly being the most classic of all, it remains useful. In addition to the aforementioned variables, the ECOG score carried greater weight. Therefore, for predicting mortality at the diagnosis of HCC, although useful for its ease of calculation and as an initial approximation, it is advisable to choose other alternatives. As for CRP/albumin, it positioned itself at a certain distance from the previous ones and at the same level as other variables, such as those dependent on the tumor.

It is important to note the presence of two isolated analytical data points that hold significant value. Firstly, elevated levels of AST hold a prominent position. This can be explained by the majority of patients exhibiting excessive alcohol consumption, a primary cause of HCC or acting as a co-factor in other conditions such as hepatitis B or C infections. In these situations, AST serves as a surrogate marker for fibrosis and acts as a strong fibrogenic factor [64,65]. Therefore, AST levels should be interpreted with caution and in an appropriate clinical context. Secondly, isolated low levels of albumin bear great importance within the developed model. As mentioned earlier, these findings may be explained by decreased albumin production in the presence of severe hepatic impairment. Additionally, these patients often exhibit a degree of associated malnutrition, both due to their hepatic damage and underlying tumor pathology [66]. 

To compare the results of this study, a focused literature search was conducted on ML. It is changing the different fields in medicine by providing sophisticated tools for data analysis, pattern recognition, and predictive modeling. In gastroenterology, ML applications have significantly improved the diagnosis, treatment, and management of gastrointestinal diseases. For instance, ML algorithms are increasingly used to analyze endoscopic images, enhance the detection of gastrointestinal lesions, and predict patient outcomes [67,68]. These advancements lead to more accurate and early diagnoses, improving patient prognosis and enabling personalized treatment plans.

Specifically in hepatology, ML has shown substantial promise in managing HCC. Various ML models, such as convolutional neural networks (CNNs) and SVM, have been developed to enhance the diagnostic accuracy of imaging techniques like ultrasound, CT, and MRI [69,70]. These models have been successful in distinguishing between different liver conditions, predicting the development of HCC, and assessing the risk of disease progression. For instance, CNN models have been trained on ultrasound images to distinguish normal liver tissues from chronic hepatitis, cirrhosis, and HCC with high accuracy [71].

Additionally, ML algorithms have been utilized to predict HCC risk using longitudinal data. These models can incorporate complex and non-linear relationships among variables, providing robust risk assessments even when traditional models like Cox regression fall short [72]. Moreover, ML models have been developed to predict the pathological grade of HCC and preoperative microvascular invasion status, which are crucial for treatment planning and prognosis [73]. Regarding the objective of our study, the use of scores that employ albumin as prognostic factors at the diagnosis of HCC yielded no results. When the search was performed based on prognostic factors for survival at the diagnosis of HCC and the use of proposed scores, few studies were found. Regarding the proposed XGB method, it is a high-performance ML algorithm known for its accuracy and efficient data handling through parallel and distributed computing. It excels in managing non-linear relationships and preventing overfitting with L1 and L2 regularization, offering flexible customization and automatic handling of missing values. The proposed XGB system also provides advanced tools for feature selection and model interpretation, along with adjustable hyperparameters and early stopping to optimize performance and minimize training time.

In the study by P-H Liu et al., it was concluded that both ALBI and PALBI were suitable models for assessing liver function and prognosis in HCC patients, with PALBI considered the superior model. They also evaluated MELD and Child-Pugh scores, both of which were deemed inferior to the previous scales [74]. Another article by Lee SK et al., assessing the utility of various scales for overall survival in HCC (including ALBI and PALBI), concluded that both PALBI and ALBI were superior to the rest of the analyzed scores (Child-Pugh and MELD) [75]. In the meta-analysis by Liu R et al., PALBI’s utility for outcomes in HCC patients with Child-Pugh A and B stages was evaluated, suggesting it might be an indicator of poor prognosis in these patients, although further studies are needed to confirm this [76]. An interesting study conducted by L-H Lu et al. PALBI was used as a predictor for post-hepatectomy liver failure and overall survival after surgery. They concluded that this score could straightforwardly predict these outcomes in patients undergoing surgical resection and classified as Child-Pugh stage A [77].

Lastly, the study by Jaruvongvanich et al. included several non-invasive tests, including ALBI, PALBI, MELD, Child-Pugh, BCLC, and others not used in our study such as the Cancer Liver Italian Program (CLIP), AST-to-Platelet Ratio Index (APRI), and Fibrosis-4 (FIB-4). Of all the studies found, this one may be the closest to the objective outlined in our study. Their conclusions indicated that CLIP was the most suitable model for predicting mortality, surpassing BCLC and PALBI, which were the next best predictors of mortality [78].

As seen in the studies found, both PALBI and ALBI were useful for this purpose, with PALBI being superior in most cases (with the special mention that in the last of them, the BCLC score was superior to both). This aligns with the conducted study where the BCLC score outperformed other analyzed metrics. The differences between PALBI and ALBI are minimal in the study conducted, which represents a significant contrast to the cited studies. Additionally, it is crucial to note the slight difference between the results of the BCLC score and both, making them an acceptable alternative for predicting mortality in these patients.

Regarding the ML methodology used, the algorithm proposed in the study, XGB, achieved the best results for all the analyzed metrics. This confirms its usefulness and reliability for the automatic classification of data in these patients, which is also corroborated by the similarity in performance between the training and test phases in the final radar plot. Therefore, this model does not lose predictive capacity. Additionally, its speed of execution and high scalability make it a useful tool for daily activities.

Alongside the inherent limitations of a retrospective study, perhaps the major limitation of this study is the total sample size. However, considering the pathology under study and its prevalence, the number of patients is not negligible. Moreover, ML techniques allow for limitations to be minimized, especially when dealing with a small sample size [79]. By optimizing hyperparameters, it’s possible to maximize the results of all the analyzed algorithms. The study was repeated 100 times to obtain statistically significant metric values and thereby reduce potential noise present in the sample [80].

## 5. Conclusions

In conclusion, PALBI and ALBI serves as a strong predictor of mortality upon HCC diagnosis, nearly comparable to the BCLC score. Due to their easy calculation, these scores can be readily employed in daily clinical practice consistent with the study’s aim. Isolated low levels of albumin also proved to be a useful tool, surpassing both the Child-Pugh score and CRP/Albumin. The XGB algorithm developed a superior model compared to the other methods used to identify the utility of key scores related to albumin in the diagnosis of HCC. This final model allowed the assessment of the commonly used scores according to scientific literature. Thanks to this model, the utility of PALBI and ALBI is confirmed, facilitating personalized decision-making for these patients.

## Figures and Tables

**Table 1:** Main hyperparameters of the machine learning algorithms evaluated in the study.

Method	Parameters
SVM	Kernel function: Gaussian Sigma = 0.5 C = 1.0 Numerical tolerance = 0.001 Iteration limit = 100
BLDA	Kernel: Bayesian
GNB	Usekernel: False fL = 0 Adjust = 0
KNN	Number of neighbours = 20 Distance metric: Euclidean Weight: Uniform
XGB	Base estimator: tree Maximum number of splits = 20 Learning rate = 0.1 Number of learners = 50

**Table 2:** Summary of values for accuracy, recall, specificity, and precision for the different analyzed Machine Learning algorithms. SVM: Support Vector Machine. BLDA: Bayesian Linear Discriminant Analysis. DT: Decision Tree. GNB: Gaussian Naïve Bayes. KNN: K-Nearest Neighbors. XGB: eXtreme Gradient Boosting.

Methods	Accuracy	Recall	Specificity	Precision
BLDA	80.25	80.35	80.16	80.67
SVM	82.45	82.54	82.35	82.86
KNN	87.64	87.75	87.54	87.03
GNB	74.12	74.21	74.03	73.59
DT	85.55	85.65	85.45	84.94
XGB	94.05	94.07	93.84	93.28

**Table 3:** Results of the statistical metrics analyzed for the assessment of the different Machine Learning implemented algorithms. AUC: Area Under the Curve. MCC: Matthews Correlation Coefficient. DYI: Youden’s Index. SVM: Support Vector Machine. BLDA: Bayesian Linear Discriminant Analysis. DT: Decision Tree. GNB: Gaussian Naïve Bayes. KNN: K-Nearest Neighbors. XGB: eXtreme Gradient Boosting.

Methods	AUC	F1 Score	MCC	DYI	Kappa
BLDA	0.80	80.01	71.10	80.25	71.34
SVM	0.83	82.20	73.16	82.45	73.40
KNN	0.87	87.38	77.88	87.64	77.14
GNB	0.74	73.90	65.77	74.12	65.99
DT	0.85	85.29	75.91	85.55	76.16
XGB	0.94	93.67	83.37	93.95	83.64

## Data Availability

Additional data can be shared on request from qualified investigators for the purposes of replicating procedures and results and for other noncommercial research purposes within the limits of the participants’ consent. Correspondence and material requests should be addressed to jorge.mateo@uclm.es. Nevertheless, the program code is available at https://github.com/J211-sys/MLXGB-HCC, accessed on 12 July 2024.
